# Primary Hyperoxaluria Diagnosed Based on Bone Marrow Biopsy in Pancytopenic Adult with End Stage Renal Disease

**DOI:** 10.1155/2015/402947

**Published:** 2015-11-08

**Authors:** Pardis Nematollahi, Fereshteh Mohammadizadeh

**Affiliations:** Department of Pathology, Faculty of Medicine, Isfahan University of Medical Sciences, Isfahan 81687 93316, Iran

## Abstract

Inborn errors of metabolism cause increase of metabolites in serum and their deposition in various organs including bone marrow. Primary hyperoxaluria (PH) is a rare inborn error in the pathway of glyoxylate metabolism which causes excessive oxalate production. The disease is characterized by widespread deposition of calcium oxalate (oxalosis) in multiple organs. Urinary tract including renal parenchyma is the initial site of deposition followed by extrarenal organs such as bone marrow. This case report introduces a 54-year-old woman with end stage renal disease presenting with debilitating fatigue and pancytopenia. The remarkable point in her past medical history was recurrent episodes of nephrolithiasis, urolithiasis, and urinary tract infection since the age of 5 years and resultant end stage renal disease in adulthood in the absence of appropriate medical evaluation and treatment. She had an unsuccessful renal transplantation with transplant failure. The patient underwent bone marrow biopsy for evaluation of pancytopenia. Microscopic study of bone marrow biopsy led to the diagnosis of primary hyperoxaluria.

## 1. Introduction

Hyperoxaluria, either primary or secondary, is a disease with increased serum levels of oxalate and resultant oxaluria. Primary hyperoxaluria (PH) is a rare inborn error of metabolism in the metabolic pathway of glyoxylate which causes excessive oxalate production [1]. The disease is characterized by widespread deposition of calcium oxalate (oxalosis) in multiple organs [2]. Secondary hyperoxaluria (SH) is an acquired disorder secondary to excessive dietary intake of oxalate Crohn's disease, chronic hemodialysis, or bowel resection that must be excluded before making the diagnosis of PH [2–4].

Oxalate when combined with calcium has a high tendency to deposit in multiple organs [3]. This condition called oxalosis is a phenomenon in which calcium oxalate crystals deposit in renal and extrarenal organs [5]. Tubulointerstitium of renal parenchyma is the first site of calcium oxalate deposition and causes both acute and chronic tubulointerstitial nephritis and also results in nephrolithiasis and consequent renal failure. Crystal deposition in kidneys is followed by deposits in bone marrow and other tissues. Diffuse replacement of marrow parenchyma by crystals leads to pancytopenia and a leukoerythroblastic reaction [6].

Herein, we report a case of primary hyperoxaluria diagnosed based on bone marrow biopsy in a 54-year-old pancytopenic woman with end stage renal disease, although the diagnosis of primary hyperoxaluria is not made usually by bone marrow biopsy.

## 2. Case Report

A 54-year-old female presented with debilitating fatigue. The remarkable point in her past medical history was recurrent episodes of bilateral nephrolithiasis, urolithiasis, and urinary tract infection since the age of five years leading to end stage renal disease (ESRD) in adulthood in the absence of appropriate medical evaluation and treatment. She also mentioned arthritis pain in knees and another joints. She had renal transplantation two years ago with unsuccessful outcome of transplant failure within the first month following surgery. At the time of admission, she was under regular hemodialysis three times a week.

On physical examination, no organomegaly was detected. Complete blood count showed hemoglobin level of 8.3 gm/dL, white blood cell count of 3.7 × 10^9^/L, and platelet count of 85 × 10^9^/L. Remarkable serum biochemistry lab data included creatinine of 5.4 mg/dL, BUN of 60 mg/dL, potassium of 5.5 mEq/L, serum oxalate level of 285 microgram/dL, and ferritin of 1990 ng/mL; liver enzymes and bilirubin are within normal limit.

**Figure 1:**
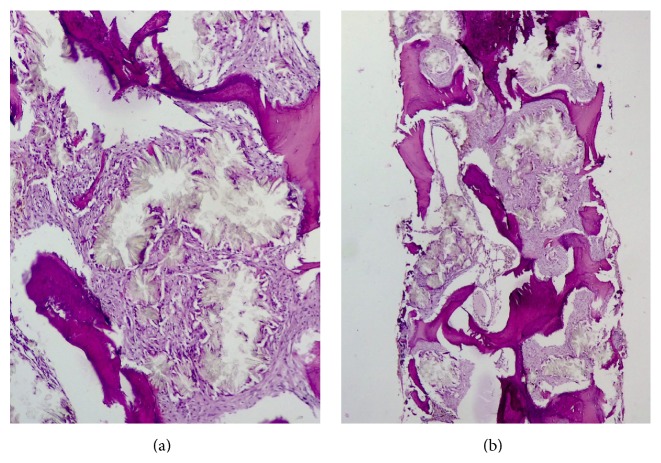
Extensive starburst crystal deposition in bone marrow, surrounded by foreign body giant cells and fibrosis. (a) ×400. (b) ×100.

Ultrasonography showed radiological findings of failure in transplanted kidney with diffuse nephrolithiasis and hydronephrosis in patient's kidneys. The patient was referred to hematology/oncology department for evaluation of pancytopenia and bone marrow studies were requested. Aspiration and touch preparation smears were dry and hypocellular, respectively. Biopsy sections showed scattered small areas of trilineage hematopoiesis with progressive maturation. There were also widespread areas of rosette-shaped arrays of intrahistiocytic needle-shaped, birefringent calcium oxalate crystals surrounded by a brisk foreign body giant cell reaction and diffuse fibrosis (Figures 1(a) and 1(b)). Although no liver biopsy or genetic studies, which is a gold standard of diagnosis testing, were done, the diagnosis of primary hyperoxaluria was made based on the characteristic morphology of crystals, renal involvement, and the absence of any secondary cause for the condition.

## 3. Discussion

In this case report, we have presented a patient with long duration history of recurrent nephrolithiasis and urolithiasis from childhood with resultant ESRD and recent debilitating fatigue. Unfortunately, recurrent nephrolithiasis of the patient had never been evaluated to find out the reason. Pancytopenia was detected during laboratory investigations for fatigue. Organomegaly was absent. The patient underwent bone marrow studies for evaluation of pancytopenia which led to the diagnosis of primary hyperoxaluria based on bone marrow biopsy findings. In general, bone marrow is an unusual route for the diagnosis of hyperoxaluria [6].

PH is a rare inborn error in the pathway of glyoxylate metabolism which leads to the overproduction of oxalate and its deposition as calcium oxalate in some organs. There are three types of PH and all are inherited through autosomal recessive pattern.

About 70% of the cases are type I PH. This type is due to defect in alanine glyoxylate aminotransferase (AGT) enzyme which is responsible for transformation of glyoxylate to glycine. Type I PH shows marked heterogeneity in expression. The age at presentation varies from less than 1 to over 50. In a case series of 155 patients the initial symptoms occurred before 1 year of age in 26% and after 15 years in 21% [1, 7]. About 10% of PH cases are included in type II disease which is due to defect in glyoxylate reductase/hydroxypyruvate reductase (GRHPR) enzyme. This enzyme converts glyoxylate to glycolate. Type II PH is a less severe disease than type I PH with milder symptoms and later onset of first presentations [8]. In a study of 13 children with type II disease, only one patient had an obvious decrease in renal function after a median four-year follow-up [9]. Type III includes about 10% of PH cases [7]. This type is due to defect in 4-hydroxy-2-oxoglutarate aldolase which causes cleavage of 4-hydroxy-2-oxoglutarate to pyruvate and glyoxylate. The mean age at presentation is 2 years and the usual presentations of pain, hematuria, and urinary tract infection are due to urolithiasis. Type III PH shows the mildest symptoms among the three types of disease and does not lead to ESRD [10].

PH is a kind of major single-gene renal disease progressing to ESRD [11]. Although type I disease is the most common type of PH, it is a rare disease in general population. Symptoms start at the median age of 5 years and initial symptoms are mostly related to urinary tract involvement [12]. Type I PH may present with variable renal presentations in different periods of life including nephrocalcinosis and renal failure in infancy, recurrent urolithiasis leading to renal failure in childhood or adolescence, occasional stone passage in adulthood, posttransplantation renal failure recurrence, and presymptomatic status with family history of PH [13]. Renal parenchyma is the first organ in which calcium oxalate deposits. Deposition of calcium oxalate is due to oversaturation of urine for this compound which leads to crystal aggregation, urolithiasis, and/or nephrolithiasis. Persistent nephrolithiasis may finally lead to ESRD. Calcium oxalate may also deposit in other organs including retina, myocardium, skin, central nervous system, and bone marrow [8]. Serum oxalate levels also increase in end stage renal disease and patient under hemodialysis.

Early diagnosis of patients affected by PH is associated with improved long term prognosis [14]. Unfortunately, diagnosis of PH is often delayed. However, there are some tests and procedures to detect suspicious patients. Stone analysis, urine oxalate measurement, and plasma oxalate determination may be helpful. Definite diagnosis of PH can be performed by liver biopsy assessment and measurement of enzyme activity and DNA detection of mutated gene. In patients with suspicious family history, genetic counseling should be considered. Prenatal diagnosis can be achieved by DNA analysis using chorionic villous biopsy samples [15].

Medical recommendations and treatments including large volume fluid intake, limitation of foods with high oxalate content, prescription of pyridoxine for converting glyoxylate to glycine, and regular dialysis to reduce serum and urine oxalate concentration are used to improve the quality of life and postpone kidney transplantation [8, 16], although dietary restrictions may not be as important for all people with primary hyperoxaluria and are mostly recommended to secondary ones [17]. This emphasizes the importance of early diagnosis as a prerequisite for successful treatment [18].

Another treatment which may act as a definitive cure is combined liver and kidney transplantation. Liver transplantation usually corrects enzyme deficiency [17, 19]. Most of the patients including the patient reported here do not respond to isolated kidney transplantation because oxalate supersaturation leads to the loss of the allograft in most cases.

In the case presented here, bone marrow oxalosis detected during the evaluation of pancytopenia led to the diagnosis of PH. There are few reports of bone marrow oxalosis associated with variable degrees of cytopenias, leukoerythroblastic reaction, and resistance to erythropoietin in English literature; some of them show marrow oxalosis [6, 19–21]. Pancytopenia resulting from oxalate crystal deposition in bone marrow is a rare complication of PH. Unfortunately, reversal of pancytopenia following transplant has been very rarely described. Sud et al. have reported the reversal of pancytopenia from bone marrow infiltration by oxalate crystals following a successful kidney transplant alone. Although combined kidney and liver transplant is the treatment of choice, a well-functioning kidney transplant is able to decrease the systemic load of oxalate and reduce the systemic complications of oxalosis [19].

Although rare, PH should be considered among the probable etiologies of every infant and child with the first stone and every adult patient with recurrent stones especially before any kind of transplantation. Early diagnosis and appropriate treatment may be helpful in preventing further complications such as bone marrow oxalosis and resultant marrow failure and cytopenias.
